# Construction of a risk prediction model using m6A RNA methylation regulators in prostate cancer: comprehensive bioinformatic analysis and histological validation

**DOI:** 10.1186/s12935-021-02438-1

**Published:** 2022-01-19

**Authors:** Yongjun Quan, Xiaodong Zhang, Hao Ping

**Affiliations:** 1 Department of Urology, Beijing Tongren Hospital, Capital Medical University, Beijing, 100730 China; 2 Beijing Advanced Innovation Center for Big Data-Based Precision Medicine, Beihang University & Capital Medical University, Beijing Tongren Hospital, Beijing, 100730 China; 3 Department of Urology, Beijing Chaoyang Hospital, Capital Medical University, Beijing, 100020 China

**Keywords:** Prostate cancer, N6-methyladenosine (m6A), The Cancer Genome Atlas (TCGA), Recurrence-free survival, m6Acluster, geneCluster, m6Ascore

## Abstract

**Background:**

Epigenetic reprogramming reportedly has a crucial role in prostate cancer (PCa) progression. RNA modification is a hot topic in epigenetics, and N6-methyladenosine (m6A) accounts for approximately 60% of RNA chemical modifications. The aim of this study was to evaluate the m6A modification patterns in PCa patients and construct a risk prediction model using m6A RNA regulators.

**Materials and methods:**

Analyses were based on the levels of 25 m6A regulators in The Cancer Genome Atlas (TCGA). Differentially expressed gene (DEG) and survival analyses were performed according to TCGA-PRAD clinicopathologic and follow-up information. To detect the influences of m6A regulators and their DEGs, consensus clustering analysis was performed, and tumor mutational burden (TMB) estimation and tumor microenvironment (TME) cell infiltration were assessed. mRNA levels of representative genes were verified using clinical PCa data.

**Results:**

Diverse expression patterns of m6A regulators between tumor and normal (TN) tissues were detected regarding Gleason score (GS), pathological T stage (pT), TP53 mutation, and survival comparisons, with HNRNPA2B1 and IGFBP3 being intersecting genes. HNRNPA2B1 was upregulated in advanced stages (GS > 7, pT3, HR > 1, and TP53 mutation), as verified using clinical PCa tissue. Three distinct m6A modification patterns were identified through consensus clustering analysis, but no significant difference was found among these groups in recurrence-free survival (RFS) analysis. Six DEGs of m6A clusters (m6Aclusters) were screened through univariate Cox regression analysis. MMAB and PAIAP2 were intersecting genes for the five clinical factors. MMAB, which was upregulated in PCa compared with TN, was verified using clinical PCa samples. Three distinct subgroups were established according to the 6 DEGs. Cluster A involved the most advanced stages and had the poorest RFS. The m6A score (m6Ascore) was calculated based on the 6 genes, and the low m6Ascore group showed poor RFS with a negative association with infiltration for 16 of 23 immune-related cells.

**Conclusion:**

We screened DEGs of m6Aclusters and identified 6 genes (BAIAP2, TEX264, MMAB, JAGN1, TIMM8AP1, and IMP3), with which we constructed a highly predictive model with prognostic value by dividing TCGA-PRAD into three distinct subgroups and performing m6Ascore analysis. This study helps to elucidate the integral effects of m6A modification patterns on PCa progression.

**Supplementary Information:**

The online version contains supplementary material available at 10.1186/s12935-021-02438-1.

## Introduction

Prostate cancer (PCa) is a leading malignant tumor among men [1]. PCa has primarily been treated with surgical prostatectomy or androgen deprivation therapy (ADT). However, it can become castration-resistant PCa (CRPC), and biochemical recurrence or metastasis may occur during traditional therapy, which is the main cause of cancer-specific death. Therefore, elucidating the molecular mechanisms related to PCa progression is crucial in the discovery of diagnostic biomarkers and therapeutic targets.

Epigenetic reprogramming is reported to serve a crucial role in the progression of PCa [2]. Recently, RNA modification has been regarded as a hot topic in epigenetic research, and nearly 172 different RNA modifications are present in MODOMICS [3]. Among them, N6-methyladenosine (m6A) is widespread throughout the transcriptome; indeed, m6A comprises approximately 60% of RNA chemical modifications and is present on 0.1% to 0.4% of total adenosine residues, including > 300 noncoding RNAs and 7600 mRNAs, in eukaryotes [4–6]. RNA m6A methylation regulates mRNA alternative splicing, stability, and intracellular localization, constituting the major posttranscriptional modification [7]. The formation of m6A is regulated by three categories of proteins: readers (which recognize m6A-modified sites, such as YTHDC1, YTHDC2, YTHDF1, YTHDF2, YTHDF3, HNRNPC, FMR1, LRPPRC, HNRNPA2B1, IGFBP1, IGFBP2, IGFBP3, RBMX, and ELAVL1), writers (methyltransferases, such as METTL3, METTL14, METTL16, WTAP, VIRMA (also called KIAA1429), ZC3H13, RBM15, RBM15B, and CBLL1), and erasers (demethylases, such as FTO and ALKBH5).

m6A regulatory genes are reported to participate in various carcinogenic and tumor progression processes [8, 9]. METTL3 is reported to advance PCa progression and is associated with poor prognosis by stabilizing the mRNAs of MYC, LEF1, and integrin β1 (ITGB1) by m6A methylation [10–12]. However, one study suggested that low expression of METTL3 is associated with resistance to therapy with androgen receptor antagonists via upregulation of NR5A2/LRH-1 [13]. This finding indicates the controversy and opposing functions of METTL3 in PCa. YTHDF2-induced AKT phosphorylation and MDB3B m6A modification may also promote PCa proliferation, migration, and invasion [14, 15]. FTO, an m6A demethylase, inhibits the invasion and migration of PCa cells by regulating total m6A levels [16]. Nevertheless, there are insufficient data on m6A regulators in PCa, and the role of m6A regulators remains controversial; in general, comprehensive transcriptome and genomic analysis is needed. This study fully analyzed m6A-related genes in PCa progression and prognosis.

## Materials and methods

### Data acquisition

Transcriptome profiling and single nucleotide variation data for prostate adenocarcinoma in The Cancer Genome Atlas (TCGA) were downloaded from the GDC Data Portal (https://portal.gdc.cancer.gov/). Copy number and clinical phenotype data were downloaded from the University of California Santa Cruz Xena (https://xena.ucsc.edu/). Gene expression matrices were extracted and obtained through Practical Extraction and Report Language (Perl) (version 5.34.0) and R software (4.0.3) (R Development Core Team, Vienna, Austria). The R package “RCircos” was used to generate Circos plots.

### Differentially expressed gene (DEG) analysis

mRNA levels were analyzed with TPM (transcripts per kilobase of exome per million mapped reads) data, which were transformed from the HTSeq-FPKM transcriptome profiling data of TCGA-PRAD. The R packages “limma” and “ggpurb” were then used to identify DEGs between the normal and tumor groups and for further statistical analysis. The Wilcoxon or Kruskal−Wallis test was performed to determine DEG levels, and P < 0.05 was identified as statistically significant. The R packages “clusterProfiler” and “enrichplot” were used to analyze Gene Ontology (GO) and Kyoto Encyclopedia of Genes and Genomes (KEGG) pathway enrichment.

### Survival and correlation analyses

Survival data for “biochemical_recurrence”, “days_to_first_biochemical_recurrence”, and “days_to_last_follow_up.diagnoses” were obtained from clinical phenotype data. When “days_to_first_biochemical_recurrence” was indicated, we regarded these patients as having “recurrence status”, and the time notated was used as the “recurrence follow-up time”. Other cases were regarded as having “no recurrence”, and “days_to_last_follow_up.diagnoses” was used as “recurrence_follow_up_time”. The R packages “survival” and “survminer” were used for survival analysis. Survival curves were evaluated through Kaplan–Meier and log-rank tests. Correlation analysis was performed through Pearson or Spearman correlation analysis, and a prognostic network map was drawn using the R packages “igraph”, “psych”, “reshape2”, and “RColorBrewer”. To build a PCa prognostic model using DEGs of m6A clusters, univariate Cox regression analysis was conducted, and P < 0.05 was used for later gene consensus clustering analysis.

### Consensus clustering analysis and principal component analysis (PCA)

To determine whether m6A regulators are related to PCa prognosis, the cohort from TCGA was allocated into different groups based on the consensus level of m6A regulators. The process was performed using the R package “ConsensusClusterPlus” and resulted in cluster consensus and item-consensus results. The graphical output consisted of heatmaps, consensus cumulative distribution function (CDF) plots and delta area plots. The cluster number was determined through a high consistency of clusters, a low coefficient of variation, and no significant increase in the CDF curve. The chi-square test or Fisher’s exact test was used to analyze clinicopathological characteristics and clustering. The heatmap was generated through the R package “pheatmap”. Recurrence-free survival (RFS) was detected among groups using Kaplan–Meier and log-rank tests. PCA was performed to judge the fitness of the classification with the prcomp function of R software.

### Gene set variation analysis (GSVA)

GSVA is used for assessing KEGG gene set enrichment, which is a nonparametric and unsupervised method [17]. GSVA could comprehensively score the gene sets of interest and translate them into the pathway level. In this study, to evaluate the potential pathway changes of different clusters, we downloaded “KEGG gene sets as Gene Symbols” from the GSEA website (http://www.gsea-msigdb.org/gsea/) and used the GSVA algorithm to comprehensively score each gene set.

### Tumor mutational burden (TMB) estimation

TMB was calculated by the total number of mutated/total covered bases [18]. PCa was classified into two groups based on the optimum threshold segmentation of TMB status population. We analyzed the relationship between the m6A score (shown as m6Ascore below) and the TMB and then performed survival analysis comparing prognosis between the high TMB and low TMB groups.

### Tumor microenvironment (TME) cell infiltration

TME infiltration levels were calculated through single-sample gene set enrichment analysis (ssGSEA) and quantified using enrichment scores [17]. The gene set of each TME infiltrating immune cell type was obtained as previously reported [19]. Correlation analysis between m6Ascore and immune-associated genes was performed to illustrate the relationship.

### Clinical PCa samples

Forty-five pathologically diagnosed PCa patients (15 with Gleason score (GS) < 7, 15 with GS = 7, and 15 with GS > 7) were recruited from Beijing Tongren Hospital and Beijing Chaoyang Hospital in accordance with the Ethics Committee of Beijing Tongren Hospital and Beijing Chaoyang Hospital, affiliated with Capital Medical University. All patients underwent prostatectomy between 2016 and 2021; PCa and adjacent normal tissues were removed and stored in liquid nitrogen. The clinicopathological characteristics of PCa patients are shown in Table 1.

**Table 1:** Clinicopathological characteristics of PCa patients

Clinicopathological parameters	Total (n = 45) (%)
Age	
Median (IQR)	65 (59.5–70.0)
Range (Min, Max)	52–78
< 65	22 (48.9%)
≥ 65	23 (51.1%)
Total PSA (t-PSA)	(ng/ml)
Median (IQR)	15.28 (8.61–38.30)
Range (Min, Max)	1.05–92.21
< 4 ng/ml	4 (8.9%)
4–10 ng/ml	12 (26.7%)
10–20 ng/ml	12 (26.7%)
> 20 ng/ml	17 (37.8%)
Gleason Score (GS)	
< 7	15 (33.3%)
= 7	15 (33.3%)
> 7	15 (33.3%)
Clinical T-stage	
T2a	6 (13.3%)
T2b	12 (26.7%)
T2c	15 (33.3%)
T3a or T3b	12 (26.7%)
Lymph node metastasis	
N0	30 (66.7%)
N1	15 (33.3%)
Distant metastasis	
M0 or Mx	41 (91.1%)
M1	4 (8.9%)
TNM stage	
I-II	25 (55.6%)
III-IV	20 (44.4%)
Log0.5 MMAB expression	
Median (IQR)	13.823837 (13.254977–14.245784)
Range (Min, Max)	11.968522–15.233100
Log0.5 IGFBP3 expression	
Median (IQR)	12.619003 (11.692416–13.321569)
Range (Min, Max)	10.851545–14.289084
Log0.5 HNRNPA2B1 expression	
Median (IQR)	11.927719 (11.106794–12.651593)
Range (Min, Max)	10.123230–13.496253

### Quantitative real-time PCR (qPCR) analysis

qPCR analysis was performed as previously described by our group [20, 21]. Total RNA was isolated using TRIzol™ reagent (Invitrogen), and complementary DNA (cDNA) was synthesized through One-Step gDNA Removal and cDNA Synthesis SuperMix (TransGen Biotech, Beijing, China). qPCR was performed using Top Green qPCR SuperMix (TransGen Biotech) on an SDS 7500 FAST Real-Time PCR system (Applied Biosystems, Foster City, CA, USA). GAPDH or 18S ribosomal RNA was used as an endogenous reference gene. The relevant primer sequences are shown in Additional file 1: Table S1.

### Immunophenoscore (IPS) analysis

IPS analysis was performed as previously reported [22]. IPS determines immunogenicity by referring to effector cells, immunosuppressive cells, MHC molecules and immunomodulators. IPS (ranging from 0 to 10) was calculated according to the gene expression of the representative cell types without bias using machine learning methods. The IPS results of TCGA-PRAD patients were downloaded from The Cancer Immunome Atlas (TCIA) (https://tcia.at/home).

### Statistical analysis

Statistical analyses were conducted using R software (4.0.3), SPSS software version 23 (IBM, Armonk, New York, USA), and GraphPad Prism 7.0 (GraphPad Software, La Jolla, CA, USA). Incomplete data were excluded. Chi-square or Fisher’s exact tests were used for categorical variables, and Wilcoxon or Kruskal–Wallis tests were used for continuous data. Correlations between two levels were assessed through Pearson or Spearman correlation analysis. Survival analysis was applied through the log-rank test of Kaplan–Meier survival analysis and hazard ratio (HR) with the 95% confidence interval (CI) of univariate Cox proportional hazard models. P < 0.05 was indicated as significant.

## Results

### Characteristics of m6A regulators in PCa

**Fig. 1:**
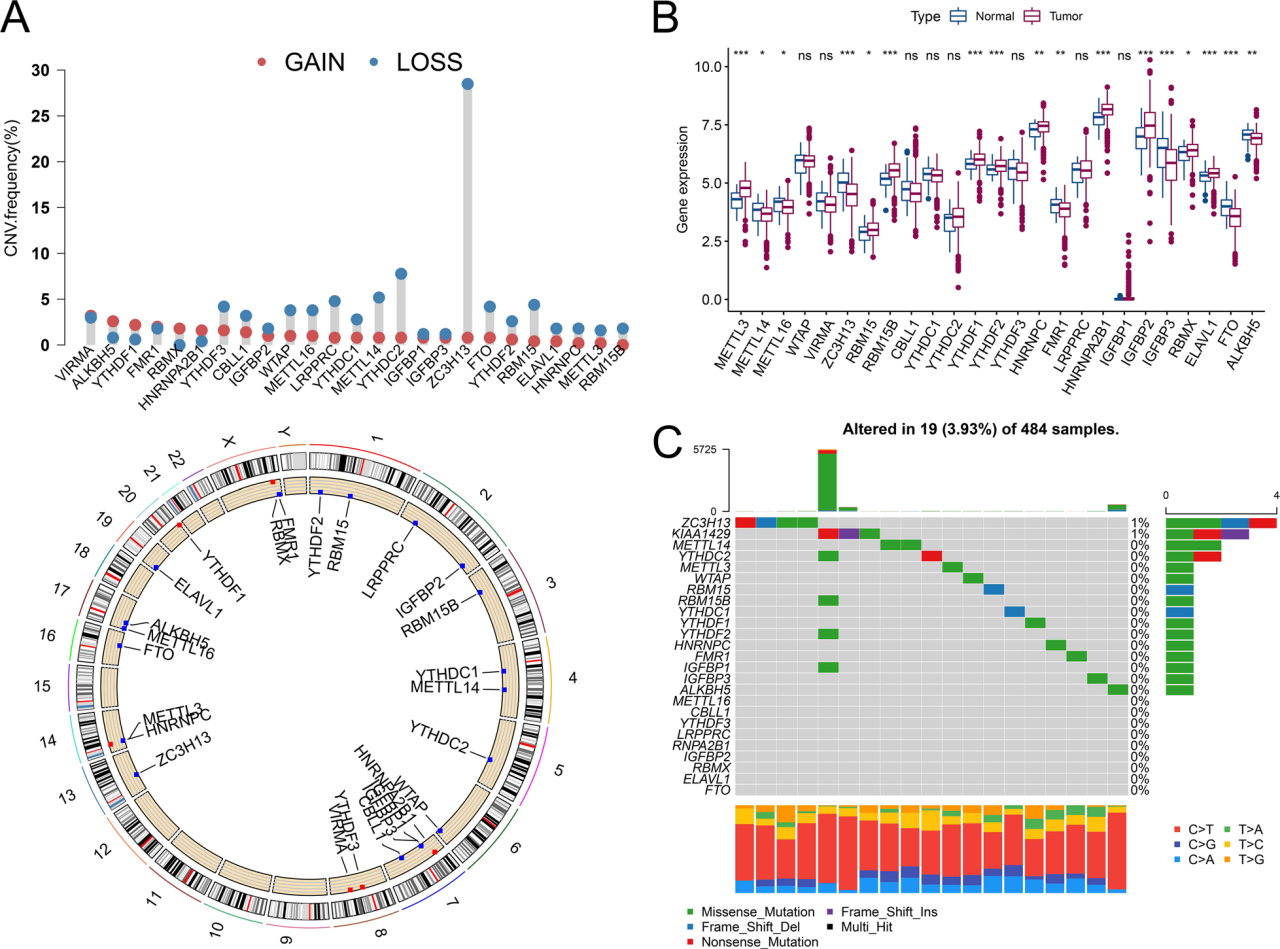
Characteristics of m6A regulators in prostate cancer. **A** (top) The CNV variation frequency of m6A regulators in TCGA-PRAD. Blue dot: deletion frequency; red dot: amplification frequency. (bottom) The location of CNV alterations of m6A regulators on 23 chromosomes. Blue dot: copy number loss, red dot: copy number gain. **B** Box plot of the expression of the 25 m6A regulators in PCa and normal tissues. Median values ± interquartile ranges are shown in the graph. ns P > 0.05; * P < 0.05; ** P < 0.01; ***P < 0.001. **C** The mutation frequency of 25 m6A regulators in 484 TCGA-PRAD patients is shown on the waterfall plot. Columns represent individual patients, the upper bar plot shows the TMB, the right bar plot shows the proportion of each variant type, and the stacked bar plot below shows the transformed fraction of each patient

In the TCGA-PRAD dataset, we analyzed copy number variation (CNV) analysis alterations, DEGs, and the mutation frequency of m6A regulators of PCa through comparison with normal samples. For CNV events, approximately 76% (19/25) of m6A regulators lost DNA copy number, with ZC3H14 having the highest degree of copy number loss (28.49%) (Fig. 1A). Six m6A regulators gained copy number, among which VIRMA had the highest such percentage (3.19%) (Fig. 1A). The m6A regulator CNV alterations and locations on chromosomes are shown in the lower panel of Fig. 1A. Of the 499 tumor and 52 normal samples in the dataset from TCGA, DEGs of m6A regulators were statistically estimated using TPM data. The levels of METTL3, HNRNPA2B1, RBM15B, and IGFBP2 were higher, while those of ZC3H13, FTO, and IGFBP3 were lower in PCa tissues than in normal tissues (P < 0.001) (Fig. 1B and Table 2). In mutation frequency analyses, 16 m6A-related genes were mutated among 19 of 484 (3.93%) samples; mutations in VIRMA (KIAA1429), YTHDC2, RBM15B, YTHDF2, and IGFBP1 were detected in one sample (Fig. 1C). ZC3H14 exhibited the highest mutation rate (4/484), and all 25 m6A regulators exhibited low mutation rates (< 1%) in TCGA samples. This suggests the relatively conserved and stable expression of m6A regulators during PCa progression.

**Table 2:** The expression levels of 25 m6A regulators in TCGA-PRAD and normal tissues

Gene	Normal (median)	Tumor (median)	P value	P symbol^a^
METTL3	4.304684	4.790198	2.44E-11	***
METTL14	3.851525	3.682336	0.03669	*
METTL16	4.198874	3.963548	0.023015	*
WTAP	5.979951	5.95777	0.740731	ns
VIRMA	4.214773	4.064446	0.255423	ns
ZC3H13	5.0224	4.529186	4.93E-08	***
RBM15	2.901797	2.98326	0.025136	*
RBM15B	5.183314	5.548273	2.44E-09	***
CBLL1	4.733786	4.543428	0.15531	ns
YTHDC1	5.389822	5.331527	0.261206	ns
YTHDC2	3.509213	3.550773	0.057348	ns
YTHDF1	5.819509	6.009969	0.000426	***
YTHDF2	5.58442	5.729154	0.000836	***
YTHDF3	5.628178	5.457101	0.396418	ns
HNRNPC	7.307854	7.455818	0.008174	**
FMR1	4.07059	3.89702	0.002945	**
LRPPRC	5.580982	5.53787	0.397438	ns
HNRNPA2B1	7.831312	8.167939	3.34E-10	***
IGFBP1	0	0	0.187698	ns
IGFBP2	6.997535	7.466738	7.00E-07	***
IGFBP3	6.508928	5.863846	7.73E-05	***
RBMX	6.32992	6.408794	0.0266	*
ELAVL1	5.323377	5.421104	0.000426	***
FTO	3.998101	3.576431	1.24E-07	***
ALKBH5	7.08196	6.930204	0.003862	**

^a^ ns P > 0.05; * P < 0.05; ** P < 0.01; ***P < 0.001

### Expression of m6A regulators in PCa prognosis and different clinicopathological characteristics

**Fig. 2:**
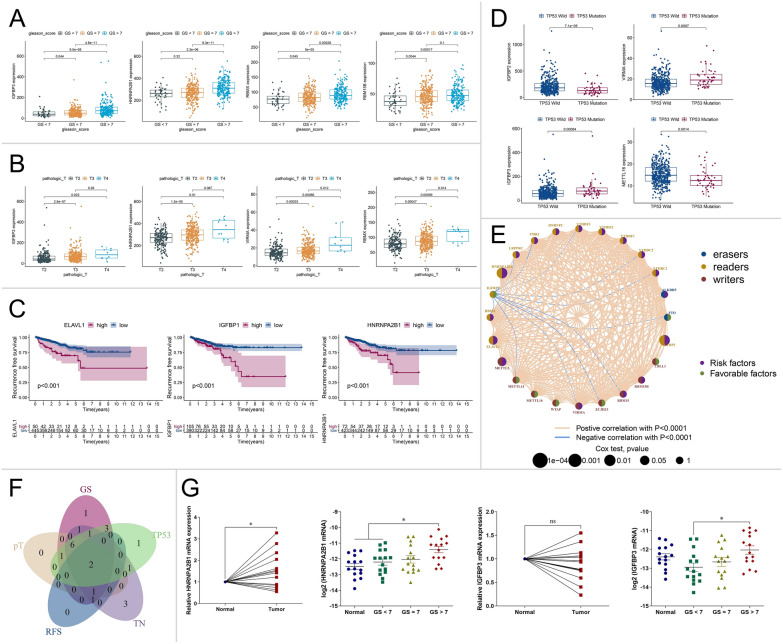
Expression of m6A regulators in relation to different PCa clinicopathological characteristics and prognoses. **A**–**D** Distribution of 3–4 representative m6A regulators (3–4 with the lowest P values) in TCGA-PRAD data stratified by GS (**A**), pT (**B**), RFS (**C**), and TP53 mutation (**D**). The box plots show the median ± interquartile range of values, and P values are presented above each pair of comparisons. **E** Prognosis network of interactions between m6A regulators in PCa. The P values of each regulator with respect to the prognosis are shown as circles of different sizes. Purple in the right hemisphere: risk factors for RFS; green in the right hemisphere: favorable factors for RFS. The erasers, readers, and writers of the m6A regulator are shown as blue, orange, and red colors, respectively on the left. Positive or negative correlations of m6A regulators are linked with lines of different colors (pink: positive, blue: negative), and the correlation strength between them is shown as different line thicknesses. **F** Intersecting DEGs of 25 m6A regulators in GS (GS > 7 vs. GS < 7), pT (T3 vs. T2), RFS (P value of univariate Cox regression analysis < 0.05), TN (tumor vs. normal PCa tissues), and TP53 (mutation vs. wild type) are shown as Venn diagrams. **G** PCa tissues and adjacent normal tissues were divided into four groups according to GS score (15 of normal tissue, 15 of GS < 7, 15 of GS = 7, and 15 of GS > 7). mRNA levels of intersecting genes were compared in different groups of PCa and adjacent normal tissues (PCa vs. adjacent normal; adjacent normal vs. GS < 7 vs. GS = 7 vs. GS > 7). Relative HNRNPA2B1 and IGFBP3 mRNA levels were assessed through qPCR analysis and normalized to adjacent normal tissues or those of endogenous reference genes. Means ± SEMs are shown in the graphs. ns P > 0.05 and * P < 0.05

The expression of m6A regulators in different GSs and pathological T (pT) stages was estimated using TCGA-PARD TPM data. As PCa with GS ≥ 7 is associated with worse prognosis [23, 24], the PCa samples were divided into three groups: GS < 7, GS = 7, and GS > 7. Compared with the GS < 7 group, IGFBP3, HNRNPA2B, RBMX, RBM15B, YTHDF1, HNRNPC, VIRMA, and FMR1 were significantly highly expressed in the GS > 7 group (P < 0.001) (Fig. 2A and Table 3). In addition, T2 (188), T3 (295), and T4 (10) pT stages were compared. Among the 25 m6A regulators, IGFBP3, HNRNPA2B1, VIRMA, and RBMX were more highly expressed in the T3 stage than in the T2 stage (P < 0.001) (Fig. 2B and Table 4). Based on Kaplan–Meier curves in survival analysis, high expression of HNRNPA2B1, IGFBP1, and ELAVL1 was associated with poor RFS (P < 0.001) (Fig. 2C). Moreover, TP53 mutation in PCa was associated with shorter radiographic progression-free survival (rPFS) and time to CRPC [25]. Compared with the TP53 wild-type group of PCa samples, the levels of VIRMA and IGFBP3 were higher and the level of IGFBP2 was lower in the TP53 mutation group (P < 0.001) (Fig. 2D and Table 5). Risk factors and relationships of m6A regulators are summarized in the prognosis network shown in Fig. 2E.

**Table 3:** Expression levels of m6A regulators in TCGA-PRAD data stratified by GS

Gene	GS < 7 (median)	GS = 7 (median)	GS > 7 (median)	P (GS < 7 vs. GS > 7)	P symbol^a^
METTL3	25.86643	26.03757	28.77871	0.012126	*
METTL14	11.3965	12.04075	11.73067	0.587863	ns
METTL16	14.60147	15.50221	14.0438	0.44366	ns
WTAP	62.4395	63.1373	57.53999	0.143969	ns
VIRMA	14.15936	15.21605	16.58136	0.000484	***
ZC3H13	23.46752	22.64276	21.06241	0.589439	ns
RBM15	6.503603	6.872581	7.042117	0.030353	*
RBM15B	37.72488	45.43605	47.3531	0.00017	***
CBLL1	23.24536	22.96488	21.80928	0.93803	ns
YTHDC1	38.39455	38.13983	40.96145	0.012687	*
YTHDC2	10.07219	10.39407	11.02557	0.050573	ns
YTHDF1	56.35877	63.87374	65.1596	0.000336	***
YTHDF2	47.1231	53.60412	52.07434	0.007882	**
YTHDF3	38.58748	43.74472	44.60192	0.009576	**
HNRNPC	148.352	171.741	180.9566	0.000415	***
FMR1	12.6015	13.62071	14.75719	0.000992	***
LRPPRC	41.37827	44.88092	47.23535	0.009325	**
HNRNPA2B1	263.0709	271.8228	310.4768	2.31E-06	***
IGFBP1	0	0	0.013463	0.024217	*
IGFBP2	187.1589	182.7438	161.6246	0.026865	*
IGFBP3	39.02542	48.8547	72.32879	9.45E-08	***
RBMX	76.6101	81.48018	88.62738	4.93E-05	***
ELAVL1	39.58125	41.91052	42.85327	0.0035	**
FTO	11.43039	11.10629	10.74576	0.705953	ns
ALKBH5	120.1901	121.221	120.7401	0.878236	ns

^a^ ns P > 0.05; * P < 0.05; ** P < 0.01; ***P < 0.001

**Table 4:** Expression levels of m6A regulators in TCGA-PRAD data stratified by pT

Gene	pT = 2 (median)	pT = 3 (median)	pT = 4 (median)	P (pT = 2 vs. pT = 3)	P symbol^a^
METTL3	27.06274	26.48476	26.30134	0.365576	ns
METTL14	11.67474	11.8227	13.97603	0.853312	ns
METTL16	15.01602	14.43229	17.49281	0.229372	ns
WTAP	62.01344	59.79612	53.04483	0.06393	ns
VIRMA	14.40579	16.51078	22.99954	0.000227	***
ZC3H13	23.14228	21.01365	22.76672	0.416107	ns
RBM15	6.749674	6.883484	8.615314	0.274807	ns
RBM15B	44.14219	46.68428	55.24486	0.0486	*
CBLL1	23.00583	21.84154	30.68415	0.360921	ns
YTHDC1	38.16906	39.80338	44.09208	0.062198	ns
YTHDC2	10.47902	10.7769	17.22446	0.439427	ns
YTHDF1	61.14875	64.5393	87.48373	0.011815	*
YTHDF2	51.36317	52.29989	68.72696	0.336839	ns
YTHDF3	41.22466	44.1152	62.55585	0.148109	ns
HNRNPC	167.5473	179.2224	204.646	0.001177	**
FMR1	13.15182	14.1732	20.4288	0.001644	**
LRPPRC	44.08828	46.08805	60.92826	0.249137	ns
HNRNPA2B1	271.8575	296.6347	344.7956	1.31E-05	***
IGFBP1	0	0	0.038231	0.100044	ns
IGFBP2	187.1589	168.3542	152.7416	0.037715	*
IGFBP3	42.2307	64.22177	83.39194	2.48E-07	***
RBMX	78.0526	86.94696	119.5574	0.000471	***
ELAVL1	40.91439	42.45284	49.47014	0.002996	**
FTO	10.76108	10.90775	13.05522	0.967256	ns
ALKBH5	121.6235	120.1459	132.7814	0.579113	ns

^a^ ns P > 0.05; * P < 0.05; ** P < 0.01; ***P < 0.001

**Table 5:** Expression levels of m6A regulators in TCGA-PRAD data stratified by TP53 mutation

Gene	Wild type (median)	Mutation (median)	P value (wild vs. mut)	P symbol^a^
METTL3	26.66187	28.77871	0.155657	ns
METTL14	11.7543	11.92501	0.210393	ns
METTL16	14.83022	12.4188	0.001348	**
WTAP	61.08309	59.07833	0.694618	ns
VIRMA	15.52701	18.9505	0.000697	***
ZC3H13	20.69471	24.60509	0.071727	ns
RBM15	6.887572	6.931969	0.381289	ns
RBM15B	46.10196	52.61076	0.008893	**
CBLL1	21.93925	24.83281	0.144508	ns
YTHDC1	38.77673	44.14293	0.01052	*
YTHDC2	10.51239	12.71358	0.008028	**
YTHDF1	62.76196	66.50237	0.045122	*
YTHDF2	51.76176	60.97605	0.011735	*
YTHDF3	42.92899	48.0619	0.037743	*
HNRNPC	175.4147	186.623	0.013856	*
FMR1	13.67919	15.39546	0.028109	*
LRPPRC	45.50271	55.43345	0.04845	*
HNRNPA2B1	285.6907	320.1461	0.005563	**
IGFBP1	0	0	0.328748	ns
IGFBP2	182.2817	130.7453	7.05E-05	***
IGFBP3	54.82908	76.41379	0.000933	***
RBMX	82.82156	93.84054	0.009741	**
ELAVL1	42.00761	42.3959	0.435743	ns
FTO	10.87488	9.600595	0.738732	ns
ALKBH5	119.9799	121.8514	0.551667	ns

^a^ ns P > 0.05; * P < 0.05; ** P < 0.01; ***P < 0.001

We analyzed DEGs of m6A regulators in GS (GS > 7 versus (vs.) GS < 7), pT (T3 vs. T2), RFS (P value of univariate Cox regression analysis < 0.05), TN (tumor vs. normal PCa tissues), and TP53 (mutation vs. wild type) comparisons. Intersecting genes were identified via a Venn diagram, and HNRNPA2B1 and IGFBP3 were differentially expressed in all comparisons (Fig. 2F). The level of HNRNPA2B1 was higher in the PCa group than in the normal group and high in association with advanced-stage parameters: namely, GS > 7, pT3, HR > 1, and TP53 mutation. However, the level of IGFBP3 was lower in PCa tissues but higher in the presence of the above advanced-stage indicators. This suggests that different molecular mechanisms and expression patterns may exist in PCa and throughout progression. We then assessed the expression of HNRNPA2B1 and IGFBP3 in 45 PCa tissues (including 15 with GS < 7, 15 with GS = 7, and 15 with GS > 7) and 15 adjacent normal prostate tissues. Levels of HNRNPA2B1 and IGFBP3 were higher in 66.7% (10/15, P < 0.05) and 40.0% (6/15, P > 0.05) of PCa tissues than in adjacent normal tissues, respectively, and the GS > 7 group exhibited elevated expression of HNRNPA2B1 and IGFBP3 compared with the GS < 7 group (both P < 0.05) (Fig. 2G).

### Consensus clustering analysis according to m6A regulators

**Fig. 3:**
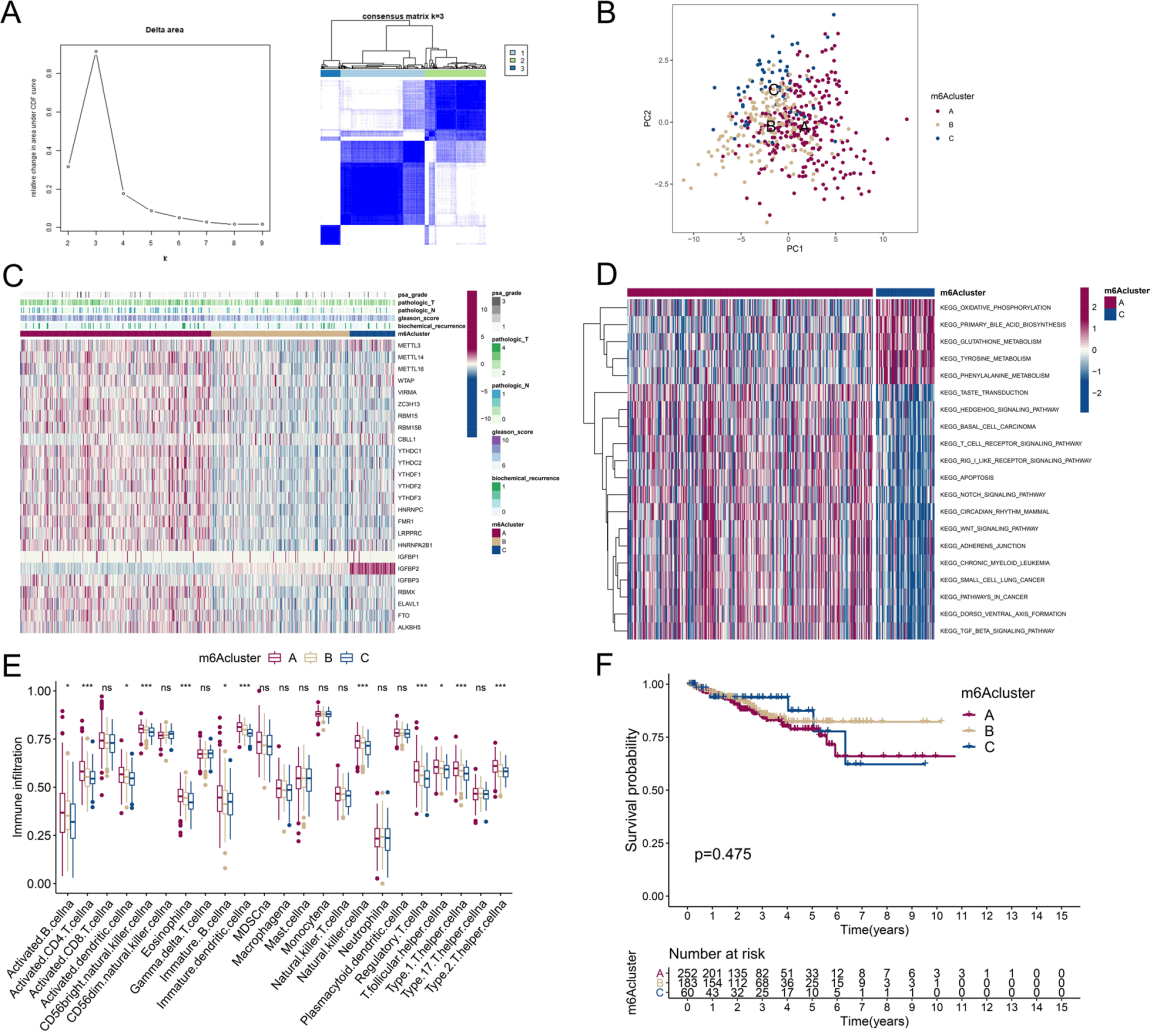
Consensus clustering analysis based on m6A regulators. **A** (left) Relative change in area under the CDF curve from k = 2 to 9. (right) Color-coded heatmap of the consensus matrix for k = 3. Color gradients represent values from 0–1 (white: 0, dark blue: 1). **B** PCA of the transcriptome profiles of three m6Acluster patterns, showing a marked difference in the transcriptome between different m6Aclusters. Different dots of red, orange, and blue in the scatter diagram represent m6Aclusters A to C. **C** The heatmap shows unsupervised clustering of 25 m6A regulators in TCGA-PRAD. PSA grade, pT, pN, GS, and biochemical recurrence were used for patient annotation. Red represents high expression, and blue represents low expression. **D** Heatmap of KEGG enrichment analysis showing the activation states of biological pathways in distinct m6Aclusters. Red represents activation, and blue represents inhibition. **E** Abundance of each infiltrating immune cell among the three m6Aclusters is shown in the boxplot. The median ± interquartile range of values is shown in the graph. ns P > 0.05; * P < 0.05; ***P < 0.001. **F** Survival analysis for RFS among three m6Aclusters based on 495 TCGA-PRAD patients. Kaplan–Meier curves and log-rank P values are shown in the graph, and the numbers at risk are shown at the bottom

To further explore the phenotypes of m6A regulators in different prognoses and clinicopathological characteristics of the TCGA-PRAD cohort, we performed consensus clustering analysis to identify subgroups of 495 PARD cases according to 25 m6A regulators (m6Acluster). Relatively high consistency, a low coefficient of variation, and an appreciable increase in the area under the CDF curve were determined as the criteria of cluster number. With regard to the relative change in the area under the CDF curves for the cluster number, k = 3 was determined to be the best category number of clusters (Fig. 3A). Subclasses were evaluated via PCA, and all three clusters were significantly distinguished, especially in clusters A and C (Fig. 3B), indicating correct prediction of the m6Acluster (these clusters are shown as “clusters 1–3 or A-C”). Next, we compared different clinicopathological characteristics of PRAD among the clusters, and the results showed relatively higher pathological N (pN) stage, GS 8–10, and biochemical recurrence rates in cluster A (Fig. 3C). The different KEGG pathways between clusters A and C were analyzed through GSVA (Fig. 3D). Cancer- and apoptosis-related pathways were significantly dysregulated between clusters A and C. We also analyzed changes in immune cell infiltration in different m6Aclusters and found 12 of 23 subpopulations of immune cells to be differentially expressed in the three clusters (Fig. 3E and Table 6). Kaplan–Meier survival curves of RFS based on the m6Aclusters demonstrated no significant differences among the groups (P = 0.475) (Fig. 3F).

**Table 6:** Abundance of each infiltrating immune cell among the three m6Aclusters

Immune cell infiltration	Group A (median)	Group B (median)	Group C (median)	P value	P symbol^a^
Activated.B.cellna	0.367609	0.35241	0.320044	0.035299	*
Activated.CD4.T.cellna	0.581698	0.553239	0.544626	2.04E-07	***
Activated.CD8.T.cellna	0.741994	0.729711	0.729734	0.119033	ns
Activated.dendritic.cellna	0.566906	0.55417	0.545927	0.014999	*
CD56bright.natural.killer.cellna	0.803422	0.796865	0.786135	0.000248	***
CD56dim.natural.killer.cellna	0.768694	0.770296	0.777682	0.468408	ns
Eosinophilna	0.452763	0.44455	0.421455	0.000297	***
Gamma.delta.T.cellna	0.672574	0.672239	0.675783	0.999306	ns
Immature..B.cellna	0.446315	0.41228	0.424357	0.01113	*
Immature.dendritic.cellna	0.812085	0.79882	0.779721	1.15E-10	***
MDSCna	0.734421	0.71748	0.710971	0.169041	ns
Macrophagena	0.493925	0.484737	0.485948	0.440833	ns
Mast.cellna	0.546259	0.546121	0.546628	0.844207	ns
Monocytena	0.880654	0.87941	0.879403	0.913355	ns
Natural.killer.T.cellna	0.466253	0.464513	0.456207	0.069941	ns
Natural.killer.cellna	0.74012	0.730841	0.715254	1.39E-05	***
Neutrophilna	0.233375	0.241803	0.236327	0.692117	ns
Plasmacytoid.dendritic.cellna	0.781692	0.779419	0.777867	0.247269	ns
Regulatory.T.cellna	0.586997	0.561565	0.544363	0.000173	***
T.follicular.helper.cellna	0.604853	0.598244	0.592525	0.029061	*
Type.1.T.helper.cellna	0.597887	0.585768	0.570837	0.000293	***
Type.17.T.helper.cellna	0.466109	0.466299	0.464527	0.584038	ns
Type.2.T.helper.cellna	0.61026	0.582465	0.582235	3.01E-09	***

^a^ ns P > 0.05; * P < 0.05; ** P < 0.01; ***P < 0.001

### Consensus clustering analysis based on DEGs of m6Aclusters

**Fig. 4:**
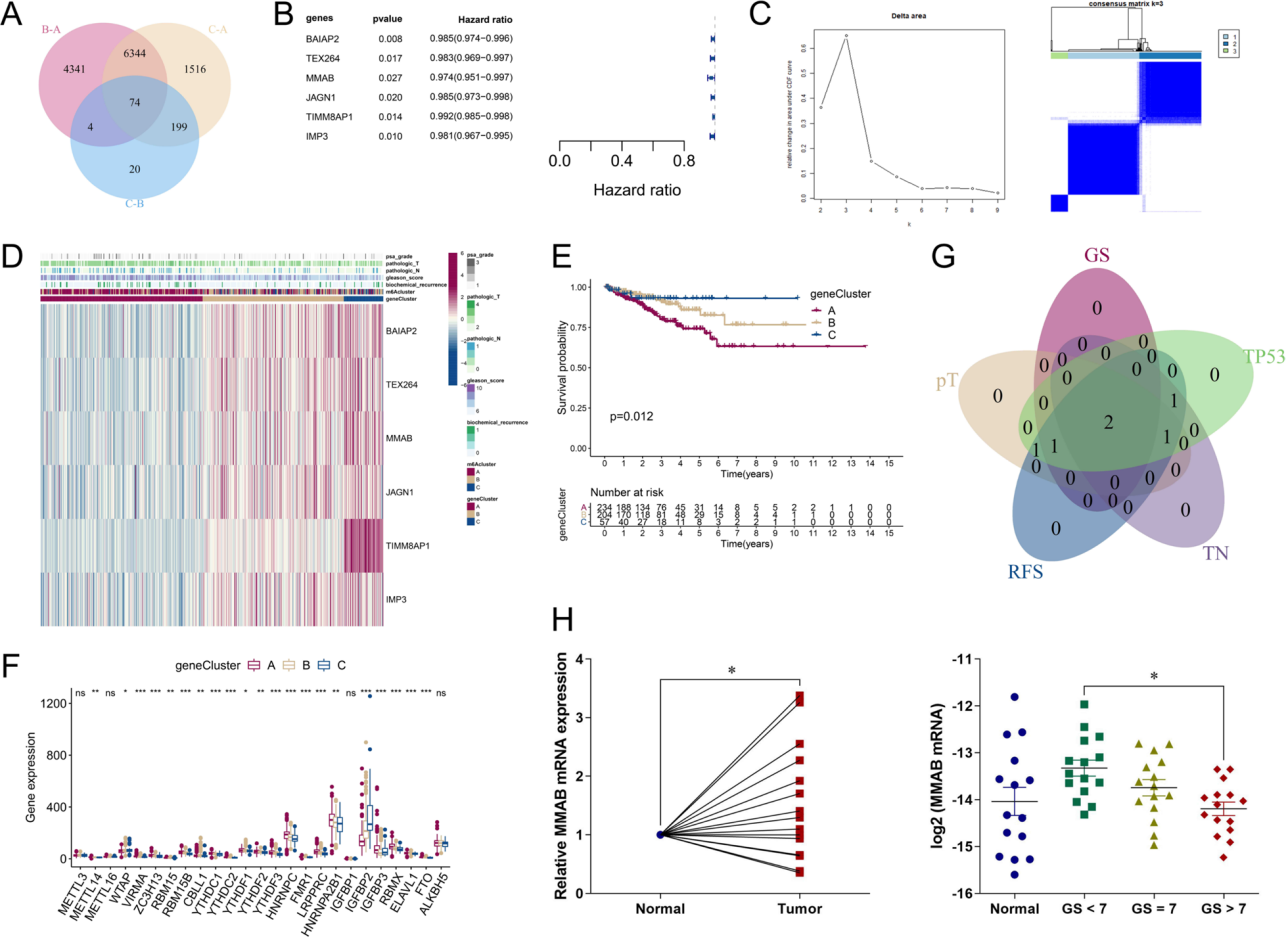
Consensus clustering analysis according to DEGs of m6Aclusters. **A** DEGs were analyzed in every pairwise comparison of m6Aclusters and intersecting genes were identified via a Venn diagram. **B** Univariate Cox regression analysis was performed to evaluate RFS in relation to 74 intersecting genes. The statistical significance (P < 0.05) of 6 genes (BAIAP2, TEX264, MMAB, JAGN1, TIMM8AP1, and IMP3), P value, and HR value (with 95% CI) are shown in the plot. **C** Consensus clustering analysis according to DEGs of m6Aclusters was performed. (left) Relative change in area under the CDF curve from k = 2 to 9. (right) Color-coded heatmap of the consensus matrix for k = 3. Color gradients represent values from 0–1 (white: 0, dark blue: 1). **D** The heatmap shows unsupervised clustering of the 6 genes in TCGA-PRAD. PSA grade, pT, pN, GS, and biochemical recurrence were used for patient annotation. **E** Survival analysis for RFS among three geneClusters based on 495 TCGA-PRAD patients. Kaplan–Meier curves and log-rank P values are shown in the graph, and the numbers of patients at risk are shown at the bottom. **F** Expression of m6A regulators among three geneClusters is shown in the boxplot. The median ± interquartile range of values is shown in the graph. ns P > 0.05; * P < 0.05; ** P < 0.01; ***P < 0.001. **G** As described in Fig. 2F, intersecting DEGs of GS, pT, RFS, TN, and TP53 are shown in a Venn diagram. **H** As described in Fig. 2G, PCa tissues and adjacent normal tissues were divided into four groups, and mRNA levels of intersecting gene (MMAB) in different groups were compared. * P < 0.05

DEGs (P value of Bayes test < 0.001) were analyzed in every pairwise comparison according to the three clusters based on m6A regulators, and the 74 intersecting genes were identified via a Venn diagram (Fig. 4A). We then analyzed the biological functions of these 74 genes, which were categorized into GO terms of biological process (BP), cell component (CC), and molecular function (MF). Under the stringent threshold of P-adjust < 0.05, only 1 specific CC (proton-transporting V-type ATPase, V0 domain) was enriched (Additional file 2: Fig. S1A-B). Additionally, KEGG signaling pathway analysis of 74 genes indicated significant enrichment in the oxidative phosphorylation KEGG pathway (Additional file 2: Fig. S1C-D).

Univariate Cox regression analysis was also performed on the 74 intersecting genes, and 6 (BAIAP2, TEX264, MMAB, JAGN1, TIMM8AP1, and IMP3) related to PCa recurrence were selected (P < 0.05) (Fig. 4B). The characteristics of the 6 or 5 DEGs (TIMM8AP1 was excluded because of being a processed pseudogene and a lack of copy number data) regarding CNV alterations, DEGs, and mutation frequency were analyzed in PCa compared with normal samples (Additional file 3: Fig. S2A-D and Additional file 1: Table S2). We found that the levels of 4 (BAIAP2, IMP3, JAGN1, MMAB) of the 6 genes were significantly upregulated in PCa tissues. Interestingly, JAGN1 exhibited the highest degree of copy number loss (5.18%), but its expression was higher in PCa tissues than in normal prostate tissues (P < 0.01). When assessing the expression of the 6 genes with respect to the clinicopathological characteristics GS, pT, and TP53, we found that most were downregulated in advanced stages (GS > 7, pT3, and TP53 mutation). Survival analysis also indicated that low expression of these 6 genes was significantly associated with poor RFS (Additional file 4: Fig. S3A-D and Additional file 1: Table S3–S5). The risk factors and relationships of the 6 genes are summarized in the prognosis network illustrated in Additional file 4: Fig. S3E.

Consensus clustering analysis was performed for these 6 genes (geneCluster), and k = 3 was determined to result in the best classification with respect to the delta area results (cluster 1–3 or A-C) (Fig. 4C). PCA verified the significance of the three subgroups (Additional file 5: Fig. S4A).

A heatmap of clinicopathological features in the three geneClusters indicated that pT stage, N stage, GS, and biological recurrence were significantly higher in geneCluster A than in the other clusters (Fig. 4D). Kaplan–Meier survival analysis revealed significant differences between the three m6Aclusters, among which geneCluster A had the poorest RFS (P = 0.012) (Fig. 4E). Next, we analyzed the expression of m6A regulators in different geneClusters and found that 21 of 25 m6A regulators were differentially expressed among them (Fig. 4F and Table 7). Moreover, 16 of 23 subpopulations of immune cells were differentially expressed among the three clusters (Additional file 5: Fig. S4B and Additional file 1: Table S6). In the GSVA of different KEGG pathways, a small cell lung cancer-related pathway was dysregulated between geneClusters A and C (Additional file 5: Fig. S4C).

**Table 7:** Expression of m6A regulators among three geneClusters

Gene	Group A (median)	Group B (median)	Group C (median)	P value	P symbol^a^
METTL3	25.43239	28.121	27.4013	0.067214	ns
METTL14	12.49848	11.61563	10.65721	0.002169	**
METTL16	15.11261	14.27289	14.50079	0.198853	ns
WTAP	59.59774	61.67926	63.11492	0.011085	*
VIRMA	17.50783	14.85196	13.20034	5.68E-09	***
ZC3H13	24.27743	19.83391	16.76071	9.62E-06	***
RBM15	7.196895	6.848082	6.450624	0.001906	**
RBM15B	50.11591	45.15223	37.2346	1.84E-09	***
CBLL1	21.41409	24.31778	19.51026	0.001104	**
YTHDC1	41.63361	37.73905	36.59234	7.15E-07	***
YTHDC2	11.26867	10.11635	8.712406	1.55E-06	***
YTHDF1	65.1071	63.04105	58.22785	0.027657	*
YTHDF2	55.0904	51.34166	48.46479	0.00317	**
YTHDF3	47.39185	42.42069	33.32456	6.17E-08	***
HNRNPC	187.0431	166.5035	152.1311	2.12E-08	***
FMR1	15.0888	13.23247	12.25827	1.79E-07	***
LRPPRC	53.01479	43.53207	37.2318	1.07E-08	***
HNRNPA2B1	299.7436	280.6586	271.21	0.001427	**
IGFBP1	0	0	0	0.057215	ns
IGFBP2	133.3923	214.2155	266.8425	2.11E-25	***
IGFBP3	66.44944	43.65073	48.12219	4.87E-08	***
RBMX	92.67909	79.35541	72.55706	1.94E-12	***
ELAVL1	44.32754	41.28624	39.84319	0.000294	***
FTO	11.70362	10.25875	7.217273	7.92E-09	***
ALKBH5	122.1352	121.0671	118.568	0.116104	ns

^a^ ns P > 0.05; * P < 0.05; ** P < 0.01; ***P < 0.001

We also analyzed DEGs of the 6 genes regarding GS, pT, RFS, TN, and TP53, as illustrated in Fig. 2G. The intersecting genes MMAB and BAIAP2 were identified via a Venn diagram (Fig. 4G), and both were highly expressed in PCa tissues compared with normal tissue but were low with respect to the four parameters, indicating advanced stage. This suggests opposite expression patterns of MMAB and PAIAP2 in PCa occurrence and progression. The mRNA expression level of MMAB was analyzed through qPCR using 45 PCa tissues and 15 adjacent normal prostate tissues, as mentioned above. It was upregulated in 60.0% (9/15, P < 0.05) of PCa tissues compared with adjacent normal tissues, with the GS > 7 group exhibiting reduced expression compared with the GS < 7 group (P < 0.05) (Fig. 4H). The differential expression of MMAB in PCa occurrence and progression was histologically verified.

### Low m6Ascore based on the 6 genes was associated with poor prognosis in RFS

**Fig. 5:**
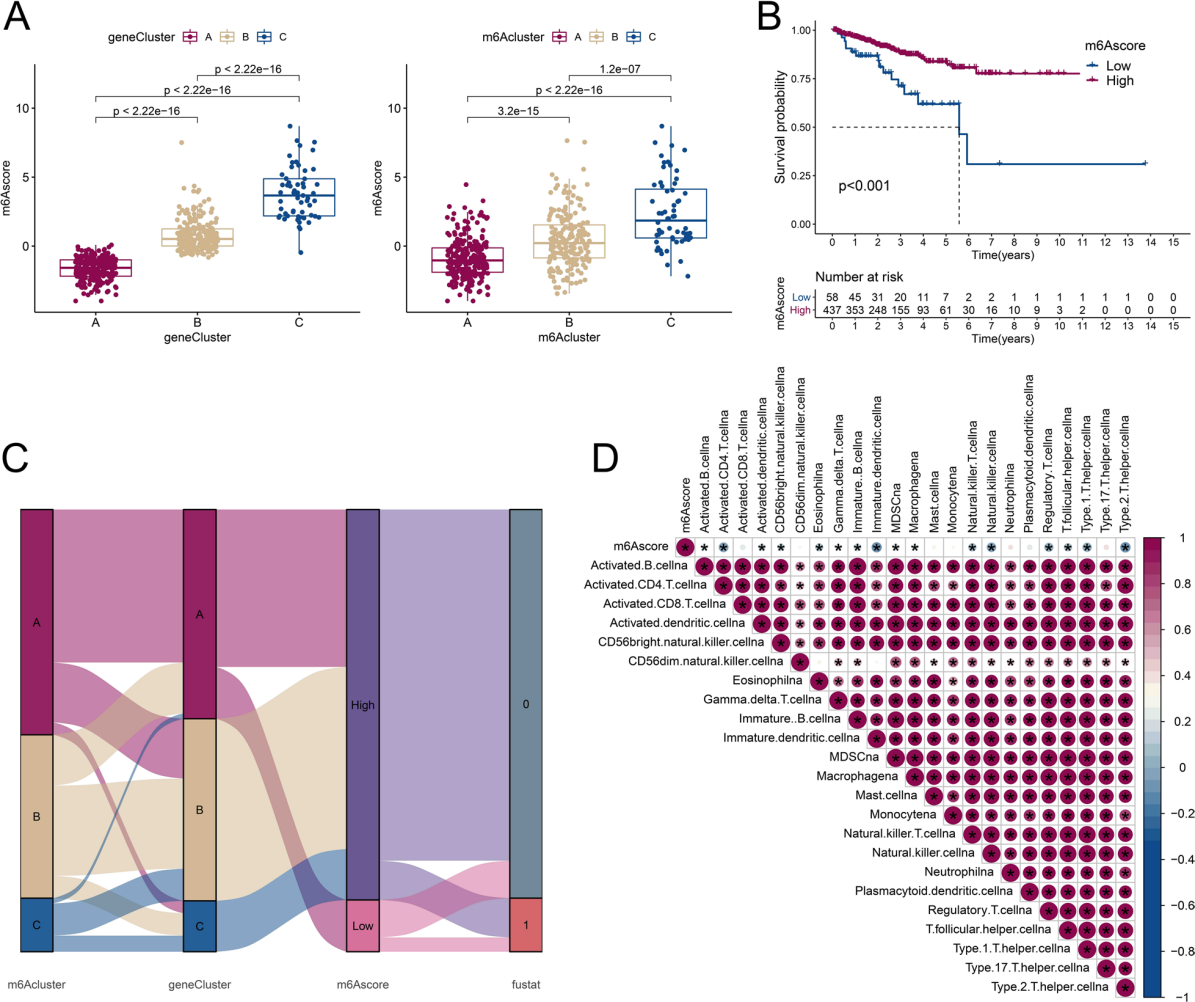
The low m6Ascore group based on the 6 genes was associated with poor prognosis in RFS. **A** The m6Ascore was calculated through PCA of the 6 gene levels in TCGA-PARD, and m6Ascores among geneClusters (left) and m6Aclusters (right) were compared. The median ± interquartile range of values and P values are shown in the box plots. **B** PRAD patients were divided into low and high m6Ascore groups based on the optimum threshold segmentation of m6Ascores in relation to RFS. The prognosis of the two groups was evaluated through survival analysis of Kaplan–Meier curve and log-rank P value. **C** Alluvial diagram showing changes in m6Aclusters, geneClusters, m6Ascore, and recurrence status (fustat of 0: no biochemical recurrence, 1: biochemical recurrence). **D** Correlations between m6Ascore and immune cell infiltration in TCGA-PRAD. Red: positive correlation, blue: negative correlation

The clustering analysis above was based on the population of the patient and could not be used to quantitatively assess the patterns of m6A regulators. Considering the complexity of m6A regulators and individual heterogeneity, the m6Ascore was calculated through PCA of the 6 gene levels in TCGA-PARD, and statistical analysis was performed to examine the relationship between m6Ascore and geneCluster/m6Acluster. The results revealed a significant difference in both clusters: geneCluster A had the lowest m6Ascore, and geneCluster C had the highest (Fig. 5A). PRAD patients were divided into two groups based on the optimum threshold segmentation of m6Ascore in RFS analysis, and according to Kaplan–Meier survival analysis, poor RFS was associated with the low m6Ascore group (Fig. 5B). The alluvial diagram shows the attribute changes in m6Acluster, geneCluster, m6Ascore, and recurrence status (fustat) (Fig. 5C). All patients in the low m6Ascore group were classified into geneCluster A, which was relevant to the worse RFS outcome (recurrence rate: 27.6%). The correlation of m6Ascore and immune-associated genes was evaluated, resulting in 16 of 23 immune-related cell infiltrates being found to be negatively related to m6Ascore (Fig. 5D). This suggests the negative regulation of the immune reaction during m6Ascore elevation. A previous study showed that low immune infiltration was potentially related to prolonged survival [26]. Consistent with our results, the high m6Ascore group had a tendency toward favorable prognosis in terms of RFS (Fig. 5B).

### Characteristics of m6Ascore status for TCGA-PRAD tumor mutation and subtypes

**Fig. 6:**
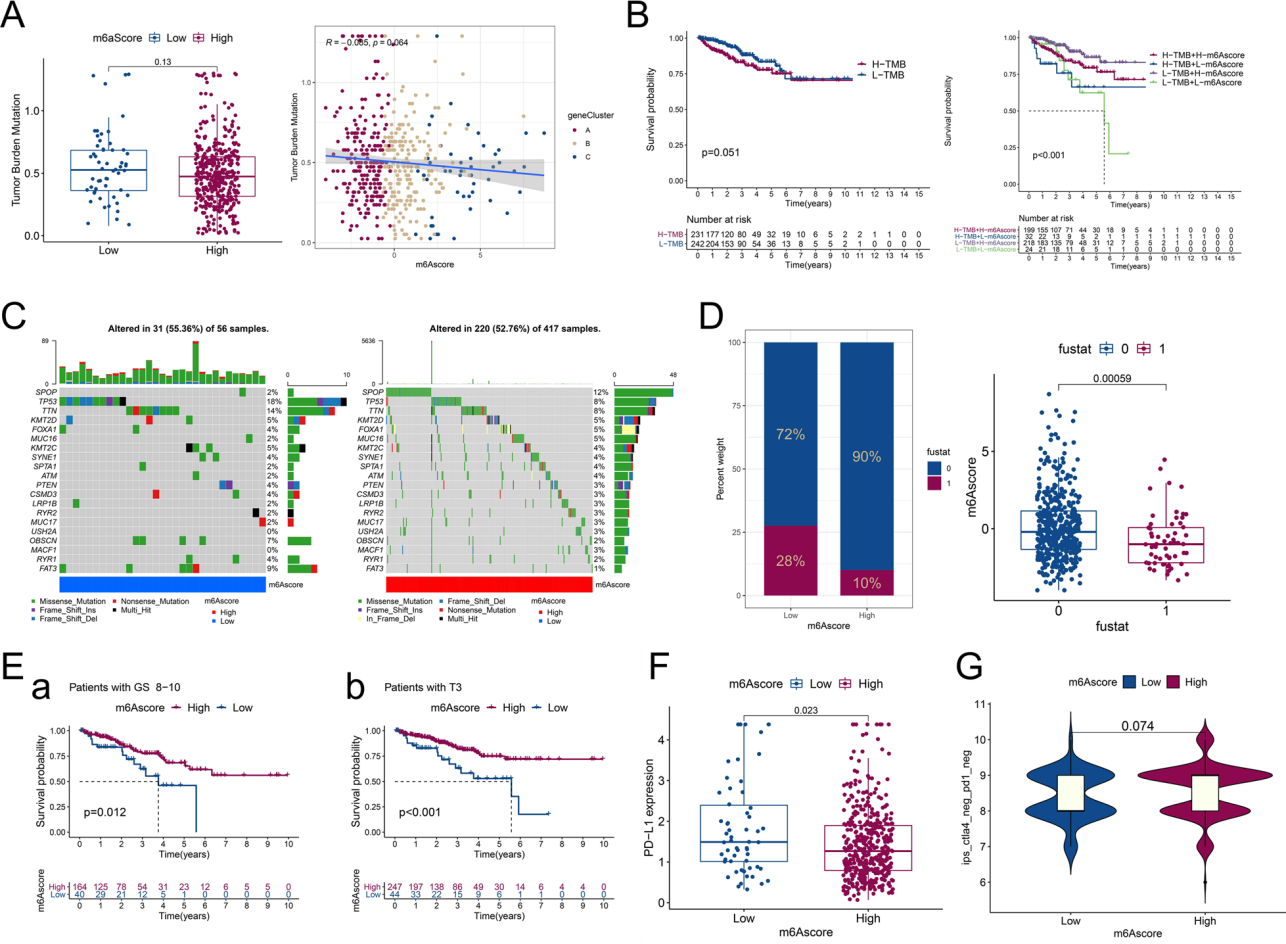
Characteristics of m6Ascore status in TCGA-PRAD tumor mutation and subtypes. **A** (left) TMB values in low and high m6Ascore groups. The P value is shown in box plot. (right) Correlation analysis of m6Ascore and TMB value in PCa was performed through Spearman correlation analysis. Different dots of red, orange, and blue in the scatter diagram represent geneClusters A to C, respectively. **B** (left) PRAD patients were divided into low and high TMB groups based on the optimum threshold segmentation of TMB values in relation to RFS. Survival analysis of the two groups is shown in the graph. (right) PRAD patients were divided into four groups based on TMB and m6Ascore status (high (H)-TMB + H-m6Ascore; H-TMB + low (L)-m6Ascore; L-TMB + H-m6Ascore; and L-TMB + L-m6Ascore), and survival analysis was performed. **C** The waterfall plot of tumor somatic mutations in PRAD with low m6Ascore (left) and high m6Ascore (right). **D** (left) The proportion of biochemical recurrence status in the m6Ascore groups (fustat of 0: no recurrence, 1: recurrence). (right) m6Ascore with respect to different biochemical recurrence statuses. The P value is shown in box plot. **E** Survival analysis of different m6Ascore groups among TRAD patients with GS 8–10 (left) and pT3 (right). **F** Box plot of PD-L1 expression in the low and high m6Ascore groups. The P value is shown in box plot. (G) Violin plot of IPS scores in ips_ctla4_neg_pd1_neg (CTLA4 negative response and PD1 negative response) in respective m6Ascore groups. The P value is shown in violin plot

A study suggested that the TMB score was positively associated with tumor-related oncogenic mutations, which led to tumor progression but was easier to detect by immune cells, making them more likely to be sensitive to immunotherapy [27, 28]. As shown in Fig. 5B, TCGA-RRAD patients were divided into two groups (58 in the low m6Ascore group and 437 in the high m6Ascore group), and the association of m6Ascore and TMB was assessed. The results showed no significant difference in TMB scores in the m6Ascore groups (P = 0.13) (Fig. 6A, left). Spearman correlation analysis also showed no significant correlation between the expression levels of m6Ascore and TMB (P > 0.05) (Fig. 6A, right). This is discordant with other study in gastric cancer, which showed that m6Ascore and TMB exhibited a significant negative correlation [19]. The PRAD patients were then divided into two groups based on the optimum threshold segmentation of TMB value in RFS analysis (242 in the low TMB group and 231 in the high TMB group). In Kaplan–Meier analysis, a tendency toward poor RFS was found in the high TMB group compared with the low TMB group, but the difference was not significant (P = 0.051) (Fig. 6B, left). This suggests that the higher malignancy of PCa patients potentially correlated with the high TMB score group. When analyzing m6A and TMB together, we found a great RFS advantage for the combination of low TMB with high m6Ascore (Fig. 6B, right). Consistent with our previous result, the low TMB and high m6Ascore groups had better prognoses in terms of RFS (Fig. 5B and Fig. 6B, left).

The distribution differences of somatic mutations were analyzed between m6Ascore groups, and the results indicated a more extensive mutation frequency of TP53 (18% vs. 8%) but a reduced mutation frequency of SPOP (2% vs. 12%) in the low m6Ascore group (Fig. 6C).

For the relationship between m6Ascore and fustat, the low m6Ascore group experienced a higher rate of recurrence (Fig. 6D left), which was consistent with the RFS analysis in Fig. 5B. Biochemical recurrence was also associated with lower m6Ascore (Fig. 6D, right). We then analyzed prognosis in the m6Ascore groups with regard to different clinicopathological features through RFS analysis (Fig. 6E and Additional file 6: Fig. S5A-B). The low m6Ascore group was associated with poor RFS in GS 8–10 and pT3 stages (P < 0.05) (Fig. 6E).

The expression of PD-L1 was detected to elucidate a potential response to immunotherapy, and the low m6Ascore group showed relatively high levels of expression (*P* = 0.023) (Fig. 6F). Finally, we further predicted the value of the risk score for immune checkpoint blockade (ICB). The IPS results of TCGA-PRAD were downloaded from the TCIA website. PD1 and CTLA4 were enrolled for IPS analysis and further classified into four parts: ips_ctla4_neg_pd1_neg (CTLA4 negative response and PD1 negative response), ips_ctla4_neg_pd1_pos (CTLA4 negative response and PD1 positive response), ips_ctla4_pos_pd1_neg, and ips_ctla4_pos_pd1_pos (Fig. 6G, Additional file 6: Fig. S5C). The average IPS in the comparison of the low and high m6Ascore groups showed no significant difference in the four parts of the negative or positive response of PD1 and CTLA4 (Fig. 6G, Additional file 6: Fig. S5C). These results indicate that m6Ascore may lack efficacy in the risk score model for predicting the response to PD1 and CTLA4 therapy.

## Discussion

In this study, we comprehensively evaluated the m6A modification patterns in TCGA-PRAD and found 6 DEGs (BAIAP2, TEX264, MMAB, JAGN1, TIMM8AP1, and IMP3) based on m6Aclusters. A model of optimal clusters was established, and m6Ascore analysis was performed based on the above 6 genes.

In general, elucidating the molecular mechanism of PCa progression remains crucial. It has been reported that epigenetic reprogramming is key for PCa progression [29, 30] and that inhibition of the epigenetic regulator EZH2 might effectively overcome ADT resistance [2]. Our previous study found that epigenetically activating AR/NDRG1 signaling through histone methylation and DNA methylation significantly suppresses CRPC progression [20, 21].

Nevertheless, the effect of mRNA posttranscriptional modification in PCa is still unclear. m6A RNA methylation may be among the most extensive RNA modifications [31–33]. Yabing Chen et al. highlighted that total RNA m6A modification levels are significantly increased in PCa tissues due to upregulation of METTL3 [34]. In addition, knockdown of METTL3 significantly reduces PCa cell migration and invasion. YTHDF2, a reader of m6A modification, synergistically induces PCa progression with METTL3 by regulating AKT phosphorylation [15]. However, low levels of METTL3 were also found to lead to advanced metastatic PCa that is resistant to androgen receptor antagonists [35]. These controversial results reveal the complexity of m6A regulators and individual heterogeneity of m6A regulators in PCa, and various m6A regulators may form a complex network structure and interact with each other to affect PCa progression.

In this study, we examined three representative groups of m6A regulators: 14 “readers”, 9 “writers”, and 2 “erasers”. Bioinformatic analysis was then performed to comprehensively investigate the association between m6A modification patterns and PCa clinicopathological characteristics and prognosis.

Compared with normal prostate tissues in TCGA database, METTL3 was significantly highly expressed in PCa, and negligible CNV and mutation rate were found in this analysis. ZC3H13 exhibited the highest degree of copy number loss (28.49%) and highest mutation rate (4/484) among all 25 m6A regulators. The mRNA of ZC3H13 was also expressed at low levels in the TN comparison (P < 0.001). Therefore, a potential mechanism including ZC3H13 mutation may exist in PCa progression. Insufficient study has been conducted to elucidate this point, which warrants further verification.

In survival and Cox regression analyses, we evaluated RFS instead of overall survival (OS) because only 10 of 493 TCGA-PARD patients died, making it difficult to obtain statistically significant results by assessing OS. In our RFS analysis, “biochemical_recurrence” and “days_to_first_biochemical_recurrence” data did not coincide, and we ultimately used the “days_to_first_biochemical_recurrence” data to confirm “recurrence status” and “recurrence follow-up time”.

The landscape of m6A variation in PCa was recently reported [26, 36–40], although no scholars have analyzed sufficient m6A regulators and verified them using clinical PCa tissues. To overcome these shortcomings, we explored all 25 widely-acknowledged m6A regulators and assessed relevant associations of various clinicopathological characteristics, such as TN, GS, pT, TP53 mutation, and survival analysis of RFS using TCGA-PRAD data. Additionally, we analyzed DEGs of m6A regulators in relation to the five factors, and the intersecting genes (HNRNPA2B1 and IGFBP3) were verified with clinical PCa tissues. The level of HNRNPA2B1 was higher in PCa tissues than in normal prostate tissues and was highly correlated with the other four advanced factors. However, the level of IGFBP3 was lower in PCa tissues but higher in the presence of the above advanced-stage indicators. This suggests that independent molecular mechanisms and expression patterns exist in PCa occurrence and progression. Consistent with the bioinformatic analysis, the mRNA levels of HNRNPA2B1 and IGFBP3 were elevated in PCa tissue of the high GS group (GS > 7) compared with the low GS group (GS < 7). In the TN comparison, the expression of HNRNPA2B1 was higher in PCa, but no significant difference in the level of IGFBP3 expression was found.

It has been reported that upregulation of HNRNPA2B1 by PCAT6 promotes PCa progression and neuroendocrine differentiation [41]. HNRNPA2B1 may also be an independent prognostic factor and contribute to cancer progression [42]. Coexpression network analysis using clinical data from the GSE70768 dataset as well as quantitative proteomic mass spectrometry profiling and gene enrichment analysis using LNCaP and PC3 cell lines suggest that HNRNPA2B1 is associated with PCa progression and prognosis [43, 44]. The level of IGFBP3 in PCa is controversial, with one meta-analysis indicating that the CC genotype of the IGFBP3–202A/C polymorphism is associated with an increased risk of PCa [45–47]. Overall, tissue verification and previous studies support a certain level of accuracy of our bioinformatic analysis using TCGA-PRAD data.

To analyze the overall effects of m6A regulators in PCa, we performed consensus clustering analysis and divided the TCGA-PRAD cohort into three subgroups based on 25 m6A regulators. As no significant difference in the three clusters for RFS was observed, this cluster analysis with m6A regulators may not be suitable for inclusion in a prognostic risk prediction model.

As epigenetic regulators, posttranscriptional modification of target genes is the primary function of m6A regulators. To analyze the downstream genes of m6A regulators, we focused on the DEGs of the m6Acluster and screened six risk genes (BAIAP2, TEX264, MMAB, JAGN1, TIMM8AP1, and IMP3) through univariate Cox regression analysis. Only IMP3 has previously been reported in PCa studies, and it appears to be increased in PCa tissues and associated with higher GS, PCa metastasis, and PCa-specific survival [48–50]. Regardless, according to our bioinformatic analysis of TCGA-PRAD, the expression of IMP3 was decreased in three groups related to advanced PCa (HR (RFS) < 1, TP53 mutation < wild type, pT3 < pT2, and tumor > normal). This finding indicates discordance between TCGA-PRAD data and previous results, which warrants further confirmation.

Consensus clustering analysis was then performed based on the 6 genes to construct a risk prediction model, and TCGA-PARD patients were divided into three subgroups. In these clusters, the clinicopathological parameters of PSA grade 3 (grade 1: > 0 and < 1, grade 2: 1–10, grade 3: > 10), pT 4, pN 1, and GS > 7 were gathered in cluster A, which was related to significantly poor RFS, suggesting an accurate risk prediction model based on 6 genes. We then analyzed the DEGs of the 6 genes in correlation with the above five factors, and MMAB and BAIAP2 were identified as intersecting genes. Levels of both were higher in PCa tissues than in adjacent normal prostate tissues but lower in tissues with advanced disease factors. We next verified MMAB based on different GSs of PCa tissues and adjacent normal tissues and found that its level of mRNA was elevated in PCa tissues but decreased in the GS > 7 group, consistent with the bioinformatic analysis.

To quantitatively illustrate the m6A signature, we calculated m6Ascore according to the expression of the 6 genes in TCGA-PARD: m6Ascore correlated positively with geneCluster, and lower m6Ascore was associated with poor prognosis in RFS. Furthermore, m6Ascore was negatively correlated with 16 of 23 immune-associated cells. Thus, a potential mechanism by which the m6A signature protects against PCa progression is by negatively regulating immune cell infiltration. A high TMB score corresponds with tumor-related mutations and sensitivity to immunotherapy [27, 28], and Yue Zhao et al. reported that m6A modification in PCa may contribute to immunotherapy strategies [26]. In our study, the lower m6Ascore group tended to have a higher proportion of TP53 mutations (18%), and the high m6Ascore group tended to have a higher proportion of SPOP mutations (12%). However, no significant correlation with TMB was found in the two m6Ascore groups in our study, and there were also no differences in PD-L1 expression or IPS score with response prediction of negative or positive PD1 and CTLA4 therapy. In summary, the results of this study do not clearly support an influence of m6A modification patterns on PCa immune cell infiltration and immunotherapy.

## Conclusions

In this study, we comprehensively analyzed m6A regulators in TCGA-PRAD through bioinformatic analysis and tissue validation. First, we screened DEGs of m6Aclusters and found 6 genes (BAIAP2, TEX264, MMAB, JAGN1, TIMM8AP1, and IMP3), through which we divided PCa patients into three subgroups and calculated m6Ascore to construct a risk model with high predictive value for recurrence. This study may contribute to determination of the effects of m6A signaling on the progression and prognosis of PCa.

## Supplementary Information

**Additional file 1: Table S1.** Oligonucleotide primers of relative genes. **Table S2.** The expression levels of 6 DEGs in TCGA-PRAD and normal tissues. **Table S3.** The expression levels of 6 DEGs in TCGA-PRAD data stratified by GS. **Table S4.** The expression levels of 6 DEGs in TCGA-PRAD data stratified by pT. **Table S5.** The expression levels of 6 DEGs in TCGA-PRAD data stratified by TP53 mutation. **Table S6.** Abundance of each infiltrating immune cell among the three geneclusters.

**Additional file 2: Fig. S1.** Enrichment analysis of 74 intersecting DEGs of m6Aclusters. (A to B) Bar plot (A) and dotplot (B) of GO enrichment in cellular component terms, biological process terms and molecular function terms. (C to D) Bar plot (C) and dotplot (D) of KEGG enriched terms. BP: biological process; CC: cell component; MF: molecular function.

**Additional file 3: Fig. S2.** Characteristics of 6 DEGs of m6Aclusters in prostate cancer. (A) (top) The CNV variation frequency of 5 DEGs (TIMM8AP1 was excluded due to being a processed pseudogene and a lack of copy number data) in TCGA-PRAD. Blue dot: deletion frequency; red dot: amplification frequency. (bottom) The location of CNV alterations of 5 DEGs on 23 chromosomes. Blue dot: copy number loss, red dot: copy number gain. (B) Box plot of the expression of 6 DEGs in PCa and normal tissues. Median values ± interquartile ranges are shown in the graph. ns P > 0.05; * P < 0.05; ** P < 0.01; ***P < 0.001. (C) The mutation frequency of 6 DEGs in 484 TCGA-PRAD patients is shown on the waterfall plot. The columns represent individual patients, the upper bar plot shows the TMB, the right bar plot shows the proportion of each variant type, and the stacked bar plot below shows the transformed fraction of each patient.

**Additional file 4: Fig. S3.** Expression of 6 DEGs of m6Aclusters in PCa prognosis and different clinicopathological characteristics. (A to D) Distribution of 3–4 representative DEGs (3–4 with the lowest P values) in TCGA-PRAD data stratified by GS (A), pT (B), RFS (C), and TP53 mutation (D). In the box plots, the median ± interquartile range of values is shown in the graph, and P values are shown above each pair of comparisons. (E) Prognosis network of interactions between 6 DEGs in PCa. The P values of each regulator of the prognosis are shown as circles of different sizes. Purple in the right hemisphere: risk factors for RFS; green in the right hemisphere: favorable factors for RFS. Upregulation (Up) or no significant differences (Ns) of 6 DEGs in PCa compared with normal tissues are filled with red and gray on the left hemisphere, respectively. Positive or negative correlations of 6 DEGs are linked with lines of different colors (pink: positive, blue: negative), and the correlation strength between them is shown as lines of different thicknesses.

**Additional file 5: Fig. S4.** Consensus clustering analysis based on 6 DEGs of m6Aclusters. (A) PCA of the transcriptome profiles of three geneClusters, showing a marked difference in the transcriptome between different m6Aclusers. Different dots of red, orange, and blue in the scatter diagram represent m6Aclusters A to C. (B) Abundance of each immune cell infiltration among three geneClusters in the boxplot. The median ± interquartile range of values is shown in the graph. ns P > 0.05; * P < 0.05; ** P < 0.01; ***P < 0.001. (C) Heatmap of KEGG enrichment analysis showing the activation states of biological pathways in distinct geneClusters. Red represents activation, and blue represents inhibition.

**Additional file 6: Fig. S5.** Characteristics of m6Ascore status in TCGA-PRAD tumor subtypes. (A) Survival analysis of different m6Ascore groups among TRAD patients with GS 6 (left) and GS 7 (right). (B) Survival analysis of different m6Ascore groups among TRAD patients with pT2 (left) and pT4 (right) disease. (C) Violin plot of IPS scores among ips_ctla4_neg_pd1_pos (CTLA4 negative response and PD1 positive response) (left), ips_ctla4_pos_pd1_neg (middle), and ips_ctla4_pos_pd1_pos (right) in the respective m6Ascore groups. The P value is shown in violin plot.

## Data Availability

The data and materials supporting the conclusions of this study are included within the article and its additional files.

## Abbreviations

PCa: Prostate cancer
ADT: Androgen deprivation therapy
CRPC: Castration-resistant prostate cancer
m6A: N6-methyladenosine
TCGA: The Cancer Genome Atlas
DEG: Differentially expressed gene
GO: Gene Ontology
KEGG: Kyoto Encyclopedia of Genes and Genomes
PCA: Principal component analysis
CDF: Cumulative distribution function
RFS: Recurrence-free survival
TMB: Tumor mutational burden
ssGSEA: Single-sample gene set enrichment analysis
qPCR: Quantitative real-time PCR
IPS: Immunophenoscore
GS: Gleason score
HR: Hazard ratio
CI: Confidence interval
pT: Pathological T
pN: Pathological N
rPFS: Radiographic progression-free survival
pN: Pathological N
OS: Overall survival

## References

[1] Siegel RL, Miller KD, Jemal A (2019). Cancer statistics, 2019. CA Cancer J Clin.

[2] Xiao L, Tien JC, Vo J, Tan M, Parolia A, Zhang Y, Wang L, Qiao Y, Shukla S, Wang X, Zheng H, Su F, Jing X, Luo E, Delekta A, Juckette KM, Xu A, Cao X, Alva AS, Kim Y, MacLeod AR, Chinnaiyan AM (2018). Epigenetic reprogramming with antisense oligonucleotides enhances the effectiveness of androgen receptor inhibition in castration-resistant prostate cancer. Cancer Res.

[3] Boccaletto P, Machnicka MA, Purta E, Piatkowski P, Baginski B, Wirecki TK, de Crécy-Lagard V, Ross R, Limbach PA, Kotter A, Helm M, Bujnicki JM (2017). MODOMICS: a database of RNA modification pathways update. Nucleic Acids Res.

[4] Cantara WA, Crain PF, Rozenski J, McCloskey JA, Harris KA, Zhang X, Vendeix FA, Fabris D, Agris PF (2011). The RNA Modification Database, RNAMDB: 2011 update. Nucleic Acids Res.

[5] Meyer KD, Saletore Y, Zumbo P, Elemento O, Mason CE, Jaffrey SR (2012). Comprehensive analysis of mRNA methylation reveals enrichment in 3' UTRs and near stop codons. Cell.

[6] Dubin DT, Taylor RH (1975). The methylation state of poly A-containing messenger RNA from cultured hamster cells. Nucleic Acids Res.

[7] Maity A, Das B (2016). N6-methyladenosine modification in mRNA: machinery, function and implications for health and diseases. Febs j.

[8] Tuncel G, Kalkan R (2019). Importance of m N(6)-methyladenosine (m(6)A) RNA modification in cancer. Med Oncol.

[9] Lin S, Choe J, Du P, Triboulet R, Gregory RI (2016). The m(6)A Methyltransferase METTL3 Promotes Translation in Human Cancer Cells. Mol Cell.

[10] Ma XX, Cao ZG, Zhao SL (2020). m6A methyltransferase METTL3 promotes the progression of prostate cancer via m6A-modified LEF1. Eur Rev Med Pharmacol Sci.

[11] Yuan Y, Du Y, Wang L, Liu X (2020). The M6A methyltransferase METTL3 promotes the development and progression of prostate carcinoma via mediating MYC methylation. J Cancer.

[12] Li E, Wei B, Wang X, Kang R (2020). METTL3 enhances cell adhesion through stabilizing integrin β1 mRNA via an m6A-HuR-dependent mechanism in prostatic carcinoma. Am J Cancer Res.

[13] Cotter KA, Gallon J, Uebersax N, Rubin P, Meyer KD, Piscuoglio S, Jaffrey SR, Rubin MA (2021). Mapping of m6A and Its Regulatory Targets in Prostate Cancer Reveals a METTL3-low Induction of Therapy Resistance. Mol Cancer Res.

[14] Du C, Lv C, Feng Y, Yu S (2020). Activation of the KDM5A/miRNA-495/YTHDF2/m6A-MOB3B axis facilitates prostate cancer progression. J Exp Clin Cancer Res.

[15] Li J, Xie H, Ying Y, Chen H, Yan H, He L, Xu M, Xu X, Liang Z, Liu B, Wang X, Zheng X, Xie L (2020). YTHDF2 mediates the mRNA degradation of the tumor suppressors to induce AKT phosphorylation in N6-methyladenosine-dependent way in prostate cancer. Mol Cancer.

[16] Zhu K, Li Y, Xu Y (2021). The FTO m(6)A demethylase inhibits the invasion and migration of prostate cancer cells by regulating total m(6)A levels. Life Sci.

[17] Hänzelmann S, Castelo R, Guinney J (2013). GSVA: gene set variation analysis for microarray and RNA-seq data. BMC Bioinformatics.

[18] Chen DS, Mellman I (2017). Elements of cancer immunity and the cancer-immune set point. Nature.

[19] Zhang B, Wu Q, Li B, Wang D, Wang L, Zhou YL (2020). m(6)A regulator-mediated methylation modification patterns and tumor microenvironment infiltration characterization in gastric cancer. Mol Cancer.

[20] Quan Y, Cui Y, Wahafu W, Liu Y, Ping H, Zhang X (2020). MLL5α activates AR/NDRG1 signaling to suppress prostate cancer progression. Am J Cancer Res.

[21] Quan Y, Zhang X, Butler W, Du Z, Wang M, Liu Y, Ping H (2021). The role of N-cadherin/c-Jun/NDRG1 axis in the progression of prostate cancer. Int J Biol Sci.

[22] Jiang Q, Chen H, Tang Z, Sun J, Ruan Y, Liu F, Sun Y (2021). Stemness-related LncRNA pair signature for predicting therapy response in gastric cancer. BMC Cancer.

[23] Eggener SE, Scardino PT, Walsh PC, Han M, Partin AW, Trock BJ, Feng Z, Wood DP, Eastham JA, Yossepowitch O, Rabah DM, Kattan MW, Yu C, Klein EA, Stephenson AJ (2011). Predicting 15-year prostate cancer specific mortality after radical prostatectomy. J Urol.

[24] Kozminski MA, Tomlins S, Cole A, Singhal U, Lu L, Skolarus TA, Palapattu GS, Montgomery JS, Weizer AZ, Mehra R, Hollenbeck BK, Miller DC, He C, Feng FY, Morgan TM (2016). Standardizing the definition of adverse pathology for lower risk men undergoing radical prostatectomy. Urol Oncol.

[25] Deek MP, Van der Eecken K, Phillips R, Parikh NR, Isaacsson Velho P, Lotan TL, Kishan AU, Maurer T, Boutros PC, Hovens C, Abramowtiz M, Pollack A, Desai N, Stish B, Feng FY, Eisenberger M, Carducci M, Pienta KJ, Markowski M, Paller CJ, Antonarakis ES, Berlin A, Ost P and Tran PT. The Mutational Landscape of Metastatic Castration-sensitive Prostate Cancer: The Spectrum Theory Revisited. Eur Urol 2021; 10.1016/j.eururo.2020.12.040 PMC10262980 33419682

[26] Zhao Y, Sun H, Zheng J, Shao C (2021). Analysis of RNA m(6)A methylation regulators and tumour immune cell infiltration characterization in prostate cancer. Artif Cells Nanomed Biotechnol.

[27] Binnewies M, Roberts EW, Kersten K, Chan V, Fearon DF, Merad M, Coussens LM, Gabrilovich DI, Ostrand-Rosenberg S, Hedrick CC, Vonderheide RH, Pittet MJ, Jain RK, Zou W, Howcroft TK, Woodhouse EC, Weinberg RA, Krummel MF (2018). Understanding the tumor immune microenvironment (TIME) for effective therapy. Nat Med.

[28] Quail DF, Joyce JA (2013). Microenvironmental regulation of tumor progression and metastasis. Nat Med.

[29] Van Neste L, Groskopf J, Grizzle WE, Adams GW, DeGuenther MS, Kolettis PN, Bryant JE, Kearney GP, Kearney MC, Van Criekinge W, Gaston SM (2017). Epigenetic risk score improves prostate cancer risk assessment. Prostate.

[30] Natesan R, Aras S, Effron SS, Asangani IA (2019). Epigenetic Regulation of Chromatin in Prostate Cancer. Adv Exp Med Biol.

[31] Desrosiers R, Friderici K, Rottman F (1974). Identification of methylated nucleosides in messenger RNA from Novikoff hepatoma cells. Proc Natl Acad Sci U S A.

[32] Alarcón CR, Lee H, Goodarzi H, Halberg N, Tavazoie SF (2015). N6-methyladenosine marks primary microRNAs for processing. Nature.

[33] Patil DP, Chen CK, Pickering BF, Chow A, Jackson C, Guttman M, Jaffrey SR (2016). m(6)A RNA methylation promotes XIST-mediated transcriptional repression. Nature.

[34] Chen Y, Pan C, Wang X, Xu D, Ma Y, Hu J, Chen P, Xiang Z, Rao Q, Han X (2021). Silencing of METTL3 effectively hinders invasion and metastasis of prostate cancer cells. Theranostics.

[35] Cotter KA, Gallon J, Uebersax N, Rubin P, Meyer KD, Piscuoglio S, Jaffrey SR, Rubin MA (2021). Mapping of m(6)A and Its Regulatory Targets in Prostate Cancer Reveals a METTL3-Low Induction of Therapy Resistance. Mol Cancer Res.

[36] Zhang Q, Luan J, Song L, Wei X, Xia J, Song N (2021). Malignant Evaluation and Clinical Prognostic Values of M6A RNA Methylation Regulators in Prostate Cancer. J Cancer.

[37] Ji G, Huang C, He S, Gong Y, Song G, Li X, Zhou L (2020). Comprehensive analysis of m6A regulators prognostic value in prostate cancer. Aging (Albany NY).

[38] Ou-Yang S, Liu JH, Wang QZ (2020). Expression patterns and a prognostic model of m(6)A-associated regulators in prostate adenocarcinoma. Biomark Med.

[39] Wang J, Lin H, Zhou M, Xiang Q, Deng Y, Luo L, Liu Y, Zhu Z, Zhao Z (2020). The m6A methylation regulator-based signature for predicting the prognosis of prostate cancer. Future Oncol.

[40] Wu Q, Xie X, Huang Y, Meng S, Li Y, Wang H, Hu Y (2021). N6-methyladenosine RNA methylation regulators contribute to the progression of prostate cancer. J Cancer.

[41] Liu B, Jiang HY, Yuan T, Luo J, Zhou WD, Jiang QQ, Wu D (2021). Enzalutamide-Induced Upregulation of PCAT6 Promotes Prostate Cancer Neuroendocrine Differentiation by Regulating miR-326/HNRNPA2B1 Axis. Front Oncol.

[42] Jiang M, Lu Y, Duan D, Wang H, Man G, Kang C, Abulimiti K, Li Y (2020). Systematic Investigation of mRNA N (6)-Methyladenosine Machinery in Primary Prostate Cancer. Dis Markers.

[43] Cheng Y, Li L, Qin Z, Li X, Qi F (2020). Identification of castration-resistant prostate cancer-related hub genes using weighted gene co-expression network analysis. J Cell Mol Med.

[44] Singh AN, Sharma N (2020). Quantitative SWATH-based proteomic profiling for identification of mechanism-driven diagnostic biomarkers conferring in the progression of metastatic prostate cancer. Front Oncol.

[45] Peng Z, Andersson K, Lindholm J, Dethlefsen O, Pramana S, Pawitan Y, Nistér M, Nilsson S, Li C (2016). Improving the prediction of prostate cancer overall survival by supplementing readily available clinical data with gene expression levels of IGFBP3 and F3 in formalin-fixed paraffin embedded core needle biopsy material. PLoS ONE.

[46] Prager AJ, Peng CR, Lita E, McNally D, Kaushal A, Sproull M, Compton K, Dahut WL, Figg WD, Citrin D, Camphausen KA (2013). Urinary aHGF, IGFBP3 and OPN as diagnostic and prognostic biomarkers for prostate cancer. Biomark Med.

[47] Qie Y, Nian X, Liu X, Hu H, Zhang C, Xie L, Han R, Wu C, Xu Y (2016). Polymorphism in IGFBP3 gene is associated with prostate cancer risk: an updated meta-analysis. Onco Targets Ther.

[48] Zhang X, Wang D, Liu B, Jin X, Wang X, Pan J, Tu W, Shao Y (2020). IMP3 accelerates the progression of prostate cancer through inhibiting PTEN expression in a SMURF1-dependent way. J Exp Clin Cancer Res.

[49] Szarvas T, Tschirdewahn S, Niedworok C, Kramer G, Sevcenco S, Reis H, Shariat SF, Rübben H, vom Dorp F (2014). Prognostic value of tissue and circulating levels of IMP3 in prostate cancer. Int J Cancer.

[50] Ikenberg K, Fritzsche FR, Zuerrer-Haerdi U, Hofmann I, Hermanns T, Seifert H, Müntener M, Provenzano M, Sulser T, Behnke S, Gerhardt J, Mortezavi A, Wild P, Hofstädter F, Burger M, Moch H, Kristiansen G (2010). Insulin-like growth factor II mRNA binding protein 3 (IMP3) is overexpressed in prostate cancer and correlates with higher Gleason scores. BMC Cancer.

