# Supplementary material for: Construction of a risk prediction model using m6A RNA methylation regulators in prostate cancer: comprehensive bioinformatic analysis and histological validation
Source: Cancer Cell Int. 2022 Jan 19;22:33. doi: 10.1186/s12935-021-02438-1 (PMC8772220; doi:10.1186/s12935-021-02438-1)
Supplement: Supplementary file 1 — Additional file 1: Table S1. Oligonucleotide primers of relative genes. Table S2. The expression levels of 6 DEGs in TCGA-PRAD and normal tissues. Table S3. The expression levels of 6 DEGs in TCGA-PRAD data stratified by GS. Table S4. The expression levels of 6 DEGs in TCGA-PRAD data stratified by pT. Table S5. The expression levels of 6 DEGs in TCGA-PRAD data stratified by TP53 mutation. Table S6. Abundance of each infiltrating immune cell among the three geneclusters. [file 12935_2021_2438_MOESM1_ESM.docx]

### Additional Table S1. Oligonucleotide primers of relative genes.

| Gene | Forward | Reverse |
| --- | --- | --- |
| HNRNPA2B1 | CAGCGGCAGTTCTCACTACA | ATCCCTCATTACCACACAGTCT |
| IGFBP3 | GTCCAAGCGGGAGACAGAATAT | CCTGGGACTCAGCACATTGA |
| MMAB | AAACGGGAGACAAAGGGTTT | TGCAATGTGCACTGGATTTT |
| 18S | AAACGGCTACCACATCCA | CACCAGACTTGCCCCTCCA |
| GAPDH | TGACTTCAACAGCGACACCCA | CACCCTGTTGCTGTAGCCAAA |

### Additional Table S2. The expression levels of 6 DEGs in TCGA-PRAD and normal tissues.

| **Gene** | **Normal (median)** | **Tumor (median)** | **P value** | **P symbol^a^** |
| --- | --- | --- | --- | --- |
| **BAIAP2** | 5.394902 | 6.004887 | 4.80E-10 | *** |
| **TEX264** | 5.76871 | 5.864096 | 0.055806 | ns |
| **MMAB** | 4.656102 | 4.848693 | 0.019665 | * |
| **JAGN1** | 6.24272 | 6.393879 | 0.003601 | ** |
| **TIMM8AP1** | 5.365713 | 5.592802 | 0.31467 | ns |
| **IMP3** | 5.830635 | 6.101706 | 0.000191 | *** |

^a^ ns P > 0.05; * P < 0.05; ** P < 0.01; ***P < 0.001.

### Additional Table S3. The expression levels of 6 DEGs in TCGA-PRAD data stratified by GS.

| **Gene** | **GS < 7 (median)** | **GS = 7 (median)** | **GS > 7 (median)** | **P (GS < 7 vs. GS > 7)** | **P symbol^a^** |
| --- | --- | --- | --- | --- | --- |
| **BAIAP2** | 69.44617 | 66.35289 | 55.79258 | 0.014975 | * |
| **TEX264** | 57.1101 | 61.00954 | 53.93431 | 0.088465 | ns |
| **MMAB** | 29.59899 | 28.01384 | 26.30513 | 0.049239 | * |
| **JAGN1** | 80.24154 | 84.74938 | 79.71041 | 0.768011 | ns |
| **TIMM8AP1** | 80.21639 | 56.48227 | 35.175 | 7.72E-08 | *** |
| **IMP3** | 66.53525 | 70.14804 | 63.43205 | 0.362777 | ns |

^a^ ns P > 0.05; * P < 0.05; ***P < 0.001.

### Additional Table S4. The expression levels of 6 DEGs in TCGA-PRAD data stratified by pT.

| **Gene** | **pT = 2 (median)** | **pT = 3 (median)** | **pT = 4 (median)** | **P (pT = 2 vs. pT = 3)** | **P symbol^a^** |
| --- | --- | --- | --- | --- | --- |
| **BAIAP2** | 68.29871 | 60.14738 | 45.96445 | 3.18807E-05 | *** |
| **TEX264** | 61.24406 | 55.55647 | 56.79395 | 0.004196924 | ** |
| **MMAB** | 29.51999 | 26.22462 | 26.69342 | 0.009348802 | ** |
| **JAGN1** | 83.8266 | 81.7332 | 77.79716 | 0.448717296 | ns |
| **TIMM8AP1** | 65.38084 | 42.30699 | 25.03333 | 1.98102E-07 | *** |
| **IMP3** | 69.94019 | 65.7906 | 61.28737 | 0.017191604 | * |

^a^ ns P > 0.05; * P < 0.05; ** P < 0.01; ***P < 0.001.

### Additional Table S5. The expression levels of 6 DEGs in TCGA-PRAD data stratified by TP53 mutation.

| **Gene** | **Wild type (median)** | **Mutation (median)** | **P (wild vs. mut)** | **P symbol^a^** |
| --- | --- | --- | --- | --- |
| **BAIAP2** | 64.98585 | 49.95984 | 3.41768E-05 | *** |
| **TEX264** | 58.72216 | 50.39975 | 0.000696584 | *** |
| **MMAB** | 28.04372 | 24.37938 | 0.00119376 | ** |
| **JAGN1** | 83.74657 | 73.29121 | 0.029291114 | * |
| **TIMM8AP1** | 47.26154 | 26.78729 | 0.000527877 | *** |
| **IMP3** | 68.65237 | 60.12583 | 0.000455627 | *** |

^a^ * P < 0.05; ** P < 0.01; ***P < 0.001.

### Additional Table S6. Abundance of each infiltrating immune cell among the three geneclusters.

| **Immune cell infiltration** | **Group A (median)** | **Group B (median)** | **Group C (median)** | **P value** | **P symbol^a^** |
| --- | --- | --- | --- | --- | --- |
| **Activated.B.cellna** | 0.371674 | 0.32752 | 0.380301 | 0.006309 | ** |
| **Activated.CD4.T.cellna** | 0.589386 | 0.551286 | 0.540052 | 1.48E-08 | *** |
| **Activated.CD8.T.cellna** | 0.746656 | 0.72245 | 0.754252 | 0.013227 | * |
| **Activated.dendritic.cellna** | 0.570169 | 0.54999 | 0.548994 | 0.008088 | ** |
| **CD56bright.natural.killer.cellna** | 0.802578 | 0.796677 | 0.803431 | 0.104424 | ns |
| **CD56dim.natural.killer.cellna** | 0.772155 | 0.769158 | 0.764748 | 0.862982 | ns |
| **Eosinophilna** | 0.455044 | 0.437858 | 0.447388 | 0.000737 | *** |
| **Gamma.delta.T.cellna** | 0.676141 | 0.671057 | 0.664862 | 0.339763 | ns |
| **Immature..B.cellna** | 0.454001 | 0.411182 | 0.433171 | 0.000983 | *** |
| **Immature.dendritic.cellna** | 0.811087 | 0.798254 | 0.797729 | 7.72E-05 | *** |
| **MDSCna** | 0.739186 | 0.709995 | 0.721055 | 0.009744 | ** |
| **Macrophagena** | 0.501186 | 0.476977 | 0.485311 | 0.064456 | ns |
| **Mast.cellna** | 0.543956 | 0.54252 | 0.565301 | 0.036402 | * |
| **Monocytena** | 0.879852 | 0.880662 | 0.877676 | 0.564595 | ns |
| **Natural.killer.T.cellna** | 0.473533 | 0.455859 | 0.464478 | 0.006373 | ** |
| **Natural.killer.cellna** | 0.743412 | 0.719855 | 0.729327 | 4.47E-05 | *** |
| **Neutrophilna** | 0.226175 | 0.244433 | 0.243417 | 0.260654 | ns |
| **Plasmacytoid.dendritic.cellna** | 0.783658 | 0.778748 | 0.778449 | 0.715935 | ns |
| **Regulatory.T.cellna** | 0.593566 | 0.546689 | 0.557859 | 9.71E-06 | *** |
| **T.follicular.helper.cellna** | 0.608335 | 0.590707 | 0.598369 | 0.031943 | * |
| **Type.1.T.helper.cellna** | 0.601692 | 0.578554 | 0.580749 | 8.08E-05 | *** |
| **Type.17.T.helper.cellna** | 0.464278 | 0.46654 | 0.479157 | 0.015244 | * |
| **Type.2.T.helper.cellna** | 0.612448 | 0.589939 | 0.570972 | 3.02E-08 | *** |

^a^ ns P > 0.05; * P < 0.05; ** P < 0.01; ***P < 0.001.
